# Circulation levels of acute phase proteins pentraxin 3 and serum amyloid A in atherosclerosis have correlations with periodontal inflamed surface area

**DOI:** 10.1590/1678-7757-2017-0322

**Published:** 2018-04-18

**Authors:** Başak Temelli, Zuhal Yetkin Ay, Hasan Basri Savaş, Fatih Aksoy, Duygu Kumbul Doğuç, Ersin Uskun, Ercan Varol

**Affiliations:** 1 Süleyman Demirel University, Faculty of Dentistry, Department of Periodontology, Isparta, Turkey.; 2 Süleyman Demirel University, Faculty of Medicine, Department of Biochemistry, Isparta, Turkey.; 3 Alanya Alaaddin Keykubat University, Faculty of Medicine, Department of Medical Biochemistry, Alanya, Antalya, Turkey.; 4 Süleyman Demirel University, Faculty of Medicine, Department of Cardiology, Isparta, Turkey.; 5 Süleyman Demirel University, Faculty of Medicine, Department of Public Health, Isparta, Turkey.

**Keywords:** Periodontitis, Acute phase proteins, Coronary artery disease, Inflammation

## Abstract

**Objectives:**

One of the plausible mechanisms in the relationship between periodontitis and coronary artery disease (CAD) is the systemic inflammatory burden comprised of circulating cytokines/mediators related to periodontitis. This study aims to test the hypothesis that periodontal inflamed surface area (PISA) is correlated with higher circulating levels of acute phase reactants (APR) and pro-inflammatory cytokines/mediators and lower anti-inflammatory cytokines/mediators in CAD patients.

**Material and Methods:**

Patients aged from 30 to 75 years who underwent coronary angiography with CAD suspicion were included. Clinical periodontal parameters (probing depth - PD, clinical attachment loss, and bleeding on probing - BOP) were previously recorded and participants were divided into four groups after coronary angiography: Group 1: CAD (+) with periodontitis (n=20); Group 2: CAD (+) without periodontitis (n=20); Group 3: CAD (-) with periodontitis (n=21); Group 4: CAD (-) without periodontitis (n = 16). Serum interleukin (IL) −1, −6, −10, tumor necrosis factor (TNF)-α, serum amyloid A (SAA), pentraxin (PTX) 3, and high-sensitivity C-reactive protein (hs-CRP) levels were measured with ELISA.

**Results:**

Groups 1 and 3 showed periodontal parameter values higher than Groups 2 and 4 (p<0.0125). None of the investigated serum parameters were statistically significantly different between the study groups (p>0.0125). In CAD (-) groups (Groups 3 and 4), PISA has shown positive correlations with PTX3 and SAA (p<0.05). Age was found to predict CAD significantly according to the results of the multivariate regression analysis (Odds Ratio: 1.17; 95% Confidence Interval: 1.08-1.27; p<0.001).

**Conclusions:**

Although age was found to predict CAD significantly, the positive correlations between PISA and APR in CAD (-) groups deserve further attention, which might depend on the higher PISA values of periodontitis patients. In further studies conducted in a larger population, the stratification of age groups would provide us more accurate results.

## Introduction

Periodontitis and atherosclerotic cardiovascular diseases, coronary artery diseases (CAD), comprise a major health problem with their high prevalence and mortality rates for the latter^6^. The most biologically plausible mechanism in the relationship between periodontitis and CAD was suggested to be the entry of oral bacteria into the circulation, resulting in activation of the host inflammatory response in favor of atheroma formation^22^ ^,^ ^30^. The inflammatory nature of atherosclerosis has led to attention the focus on acute host inflammatory markers regarding their increase in circulation to predict and/or reflect the intensity of cardiovascular diseases^1^. Acute phase reactants (APR) are the earliest and most complicated reactional response given by the organism against bacterial, viral or parasitic infections, trauma, etc^1^. Syntheses of APR are regulated by cytokines/ mediators (pro-inflammatory cytokines such as interleukin (IL)-1, interferon-γ, IL-8, IL-6, IL-11, etc., and anti-inflammatory cytokines such as IL-10, IL-4, IL-13, etc.)^1^.

APR, such as the Pentraxin (PTX) family, were suggested as sensitive biomarkers to predict the development and progression of atherosclerosis^2^. Short PTX (25 kDa), such as C-reactive protein (CRP) and serum amyloid P (SAP), are mainly produced in the liver by IL-6 stimulation in response to the inflammatory stimuli; and the long PTX3 (40-40 kDa), is produced by IL-1 stimulation, tumor necrosis factor (TNF)-α or oxidized low-density lipoprotein (OxLDL)^2^. Thus, PTX3 and CRP were suggested to be associated with atherosclerotic lesions^20^. Serum amyloid A (SAA) was suggested to enhance the local effect and to be a more valuable biomolecular diagnosis for acute myocardial infarctions than the other APR^18^.

The increase of APR in circulation and in periodontal tissues of periodontitis patients was also reported by various studies^11^ ^,^ ^13^ ^,^ ^28^. This response was suggested to influence atherosclerosis within the endothelium^1^. The correlations between plasma levels of IL-6 and TNF-α and cardiovascular risk factors were also determined^17^. The decreased levels of the anti-inflammatory cytokine IL-10 in acute coronary syndrome were found to be associated with the increased cardiovascular risk and clinical instability^3^.

There is a need for a reliable measurement system that evaluates the periodontal inflammation as a continuous variable in the relationship between periodontitis and systemic diseases. The periodontal inflamed surface area (PISA) seems to be one of the possible ways to enter to this relationship, because the periodontal pocket has served as an access gate to the systemic circulation for the periodontopathogens, and the increased levels of circulating APR and cytokines/ mediators were reported in periodontitis patients when compared to healthy controls^23^. The measurement and determination of the periodontitis-related inflamed area is a research topic which has helped to clarify the association between periodontitis and systemic diseases in recent years^19^ ^,^ ^31^. However, there has been no study into the effect of periodontitis and related systemic inflammatory burden on the serum APR and pro-inflammatory/anti-inflammatory cytokine/ mediator levels in CAD patients.

Therefore, this study aims to test the hypothesis that higher PISA values show correlations with higher circulating levels of APR and pro-inflammatory cytokines/mediators and lower anti-inflammatory cytokines/mediators in CAD patients with periodontitis.

## Material and methods

### Ethical statement

This study was approved by Süleyman Demirel University Local Ethical Committee on Clinical Investigations (Date: 19.03.2014, Decision number: 38), has been performed according with the ethical standards laid down in an appropriate version of the Declaration of Helsinki (as revised in Brazil, 2013). Seventy-seven consecutive patients who underwent coronary angiography with a CAD suspicion in Süleyman Demirel University Faculty of Medicine, Department of Cardiology between June 2014 and August 2015 participated in the study. The study was based on voluntariness; informed consent forms were signed by all patients.

### Study population

The sample size was calculated considering Type I errors (0.05), target power (0.80), estimated difference between the means (δ=ΙΙ), and the standard deviation (σ=ΙΙ) considering the clinical attachment loss value of 1 mm. Although it was not the primary outcome of the study, the mean difference of 1 mm in clinical attachment loss values was considered to determine the groups to which the patients will belong. The sample size of each group was determined at a minimum of 16 in each group.

Patients recruited had coronary artery symptoms, a potential CAD diagnosis and planned to undergo coronary angiography in Süleyman Demirel University Faculty of Medicine, Department of Cardiology. Indication for coronary angiography was either the presence of typical angina or positive/equivocal results of noninvasive screening tests for myocardial ischemia. Diagnosis of and/or being treated for CAD, having diabetes mellitus, using statins, using calcium channel blockers for hypertension therapy, pregnancy, lactation, being under age of 30, having rheumatologic disease or malignant disease, used/using antiinflammatory and/or antibiotics in the last 3 months/ currently, and having undergone periodontal therapy within 6 months were the exclusion criteria. Current and past smokers were evaluated as “smokers”. The detailed medical and dental anamneses of the patients were also obtained.

### Clinical periodontal parameters

Before coronary angiography, periodontal examination was conducted by a single calibrated examiner (BT). Reproducibility of the data collected by the examiner was assessed by performing clinical attachment loss collection in five patients. Each patient was assessed twice in a visit, with 1-hour interval between assessments. The second set of recordings was masked to the first assessment. Reproducibility of data collection was determined by calculating the percentage of sites examined in which the scores were reproduced precisely or to an accuracy of 1 mm for each site. Assessment of the mean difference in the scores (kappa value 0.87 for clinical attachment loss) between visits indicated that there was no systematic bias in measurement.

In this examination, bleeding on probing (BOP), clinical attachment loss and probing depth (PD) were recorded for six sites (mesiobuccal, mediobuccal, distobuccal, mesiolingual, midlingual, distolingual) of the teeth with a Williams periodontal probe (Hu-Friedy, Chicago, IL, USA). The BOP from the six sites (mesiobuccal, mediobuccal, distobuccal, mesiolingual, midlingual, distolingual) of the teeth was recorded as present (+) or absent (-) to calculate the PISA and periodontal epithelial surface area (PESA) scores^26^. To calculate the BOP percentage, even one site with BOP was recorded as (+) for each individual tooth, and used to calculate the percentage of bleeding teeth considering the number of teeth of the dentition.

The periodontal diagnosis of patients (gingivitis and periodontitis) was made according to the criteria proposed by the 1999 International Workshop for the Classification of Periodontal Disease and Conditions^5^ ^,^ ^24^. The classification of periodontal diagnosis depending on the severity was also evaluated after the study was conducted^12^.

The recruitment of voluntary patients proceeded between June 2014 and August 2015 in the Department of Cardiology. After angiography, the patients were classified as with CAD or not. The patients in these two groups were classified as having periodontitis or not considering their periodontal recordings obtained before angiography as follows:

Group 1: Patients with CAD and periodontitis (n=20);

Group 2: Patients with CAD and without periodontitis (n=20);

Group 3: Patients without CAD and with periodontitis (n=21);

Group 4: Patients without CAD and without periodontitis (n=16).

### Coronary angiography

Coronary angiography was routinely performed without use of nitroglycerin. Selective coronary angiography was performed with Judkins technique in multiple projections. Iohexol (Omnipaque, Opakim, İstanbul, Turkey) was used as a contrast agent during coronary angiography in all patients and control patients. Coronary angiograms were analyzed by two blinded interventional cardiologists (FA and EV) without knowledge of the clinical status and laboratory measurements of patients. CAD was defined as >50% stenosis of one or more epicardial coronary artery. A normal segment was defined as a coronary artery segment without ectasia or stenosis based on coronary angiography^27^.

### Serum samples

Before coronary angiography, fasting blood samples from the antecubital vein were obtained. Blood samples were centrifuged at 4500 rpm for 5 minutes. Obtained serum samples were portioned and stored at −80°C until the analysis of the serum markers (IL-1, IL-6, IL-10, TNF-α, SAA, PTX3, hs-CRP).

### Biochemical analysis

Serum samples were thawed before analysis. Eight different concentrations were used as standards to obtain a standard absorbance curve and each test level was determined using this curve. The presence and levels of interleukin IL-1β, IL-6, IL-10 and TNF-α (eBioscience, Vienna, Austria), PTX3 and SAA (SunRed Biotechnology Company, Shanghai, China) were determined by commercial ELISA kits. The sensitivity of the kits are 0.3 pg/ml, 0.92 pg/ml, 0.13 pg/mL, 2.3 pg/ml, 1.05 μg/mL, 0.5 μg/mL, and 0.051 ng/ml, respectively. The high-sensitivity C-reactive protein (hs-CRP) was analyzed with an immunoturbidometric method (Beckmann Coulter AU5400, CA, and USA).

### Statistical analysis

The variables were investigated using visual (histograms, probability plots) and analytical methods (Kolmogorov-Smirnov/Shapiro-Wilks's test) to determine whether they are normally distributed. All variables were determined not normally distributed (p<0.05). The parameters are shown as the median (minimum-maximum values). The Kruskal-Wallis test was used to determine the between-group differences for the continuous parameters, and chi-square test was used for the categorical parameters. A p value less than 0.05 was considered to show significant results. To avoid Type-I errors, a Bonferroni correction was applied, and differences between the group pairs were investigated, using the Mann-Whitney U test, with a p value less than 0.0125, being considered significant. Spearman's Rank-order correlation test was used to define the correlations between variables. For multivariate analysis, the possible factors identified with univariate analysis were further entered into the logistic regression analysis to determine the independent predictors of CAD. Hosmer-Lemeshow goodness of fit statistics were used to assess model fit (Table 5). Statistical analysis was performed using the SPSS 15.0 statistical software (SPSS 15.0, Chicago, IL, USA).

## Results

Seventy-seven patients (30 female, 47 male) aged between 33 and 71 years participated in the study. Sociodemographic parameters and characteristics of the study population are in Table 1. Study groups were statistically similar regarding sex, smoking status, annual income, education level, and anthropometric measures, such as body mass index (BMI), waist and hip ratio (p>0.0125, Table 1). The CAD (+) groups (Group 1 and 2) are significantly older than the CAD (-) groups (Groups 3 and 4), (p<0.0125, Table 1).

**Table 1:** Characteristics of study groups [median (minimum-maximum)]

	Group 1 (n=20)	Group 2 (n=20)	Group 3 (n=21)	Group 4 (n=16)
Age	59.5 (46-68) †	57.5 (44-65) †	50 (42-71) ‡	49 (33-65) ‡
Gender (F, n)	7	6	7	10
Smoker (n)	1	4	5	0
Education (higher, n)	2	3	3	5
Income § (YTL, x1000)	13.8 (2.4-60)	13.2(8.4-48)	12 (2.4-36)	14.4 (6-48)
Height (m)	1.671 (1.5-1.8)	1.7 (1.5-1.84)	1.65 (1.5-1.8)	1.62(1.5-1.8)
Weight (kg)	75 (56-103)	77.5 (45-103)	75 (67-88)	72(60-97)
BMI (kg/m^2^)	27.021	26.99	28.04	29.341
	(18.29-40.23)	(19.2-36.06)	(21.6-34.37)	(22.08-39.11)
Waist (cm)	93.5 (65-122)	96 (61-120)	93 (78-120)	88.5 (68-115)
Hip (cm)	110 (85-120)	109 (89-114)	109 (87-125)	109.5 (94-125)
W/H	0.86 (0.08-1.1)	0.89 (0.68-1.09)	0.86 (0.72-1.09)	0.81 (0.7-1.17)
Total cholesterol (mg/dl)	186 (106-291)	164 (109-389)	171 (109-232)	182 (129-261)
LDL (mg/dl)	118 (51-347)	113 (46-400)	102 (46-193)	115 (72-166)
HDL (mg/dl)	46.5 (31-56)	42 (25-65)	46 (33-54)	46 (34-75)

YTL, New Turkish Lira;

† significant difference between CAD (+) P (+) and CAD (-) P (-);

‡ significant difference between CAD (-) P (+) and CAD (-)P (-);

§ The average national income is 25.13 (x1000) YTL, and 1YTL is 2.92 USD for that period.

Mann Whitney U test (with Bonferroni correction, p<0.0125)

The periodontal parameters, PISA and PESA values of the study groups are shown in Table 2. Group 4 showed significantly lower values of PD, clinical attachment loss, BOP, PISA, and PESA than the other three groups (p<0.0125). The groups with periodontitis (Groups 1 and 3) had higher PD, clinical attachmet loss, BOP, PISA, and PESA values than the groups without periodontitis (Groups 2 and 4), (p<0.0125, Table 1).

**Table 2:** Periodontal parameters, PISA and PESA values [median (minimum-maximum)]

	Group 1 (n=20)	Group 2 (n=20)	Group 3 (n=21)	Group 4 (n=16)	P
	CAD (+) P (+)	CAD (+) P (-)	CAD (-) P (+)	CAD (-) P (-)	
PD (mm)	3.185	2.12	3.41	1.955	<0.000
	(2.23-4.16)	(1.4-2.75)	(2.75-4.26)	(1.33-2.45)	†, ‡, §, ¶
CAL (mm)	3.59	2.125	3.73	1.99	<0.000
	(3.03-4.46)	(1.65-2.84)	(2.75-6.35)	(1.73-2.57)	†, ‡, §, ¶
BOP (%)	45.86	16.66	45.83	19.775	<0.000
	(27.01-79.48)	(6.14-45.37)	(1597-87.03)	(7.5-40.47)	†, ‡, §, ¶
PISA (mm^2^)	691.825	124.72	625.02	200.21	<0.000
	(174.04-1212.09)	(28.13-427.26)	(305.43-1787.09)	(28.13-427.11)	†, ‡, §, ¶
PESA (mm^2^)	1526.07	944.37	1434.46	875.205	<0.000
	(563.82-2554.74)	(326.31-1225.1)	(598.93-2398.92)	(674.83-1224.7)	†, ‡, §, ¶

† significant difference between CAD (+) P (+) and CAD (-) P (-);

‡ significant difference between CAD (-) P (+) and CAD (-) P (-);

§ significant difference between CAD (+) P (+) and CAD (+) P (-);

¶ significant difference between CAD (+) P (-) and CAD (-) P (+);

Mann-Whitney U test (with Bonferroni correction, p<0.0125)

The serum cytokine and APR levels are in Table 3. None of the investigated parameters were statistically significantly different between the study groups (p>0.0125).

**Table 3:** Serum cytokine and acute phase reactants (APR) levels [median (minimum-maximum)]

	Group 1 (n=20)	Group 2 (n=20)	Group 3 (n=21)	Group 4 (n=16)
	CAD (+) P (+)	CAD (+) P (-)	CAD (-) P (+)	CAD (-) P (-)
IL-1β (pg/mL)	8.20 (3.80-24.00)	7.50 (3.40-11.10)	8.00 (3.20-18.00)	2.00 (3.50-11.00)
IL-6 (pg/mL)	2.20 (1.00-5.80)	2.10 (1.00-6.08)	2.00 (0.90-3.50)	0.50 (0.90-2.80)
IL-10 (pg/mL)	1.20 (0.10-7.00)	1.30 (0.08-3.80)	1.20 (0.02-3.20)	0.60 (0.90-2.30)
TNF-α (pg/mL)	0.50 (0.02-2.00)	0.40 (0.08-0.88)	0.50 (0.08-1.60)	0.30 (0.03-0.90)
hs-CRP (mg/L)	2.80 (0.90-18.00)	5.60 (0.90-49.40)	1.50 (0.90-2.90)	2.90 (0.90-12.15)
PTX3 (pg/mL)	2.90 (0.70-7.00)	1.40 (1.40-7.90)	2.00 (1.30-3.05)	1.10 (0.90-4.85)
SAA (μg/mL)	2.20 (0.10-7.10)	1.70 (0.40-4.20)	2.00 (0.60-5.18)	1.20 (0.20-4.60)

Mann-Whitney U test (with Bonferroni correction, p<0.0125)

In Table 4, the statistically significant correlations between the investigated parameters are shown. Correlations between the parameters were tested in groups with CAD (Groups 1 and 2, n=40), in groups without CAD (Groups 3 and 4, n = 37), and in the whole group (N = 77). In the CAD (+) groups (Groups 1 and 2), PD showed a significant negative correlation with serum IL-10 level (p<0.05). In CAD (-) groups (Groups 3 and 4), the PISA value showed significant correlations with PTX3 and SAA (p<0.05).

**Table 4:** Statistically significant correlations between serum cytokine and APR levels, systemic inflammatory burden and clinical periodontal parameters

Groups	Parameters	r	^p^
CAD (+) P (+/-)	PD-IL-10	0.453	0.040*
(Group 1+2, n= 40)			
CAD (-) P (+/-)	PISA-SAA	0.453	0.045*
(Group 3+4, n= 37)	PISA-PTX3	0.635	0.003*
	PD-TNF-α	0.482	0.032*
Whole group (N=77)	PD-IL-10	0.24	0.035*
	BOP-TNF-α	0.276	0.015*

r, Pearson Correlation coefficient (Pearson's correlation analysis, *p<0.05)

In Table 5, the predictability of the variables for CAD was investigated. Age was found to predict CAD significantly according to the results of the multivariate regression analysis (Odds ratio: 1.17; 95% Confidence interval: 1.08-1.27; p<0.001) (Hosmer-Lemeshow test, p=0.150).

**Table 5:** Odds ratios (OR) and confidence intervals (CI) of multivariate logistic regression model [Factors associated with having coronary artery disease (CAD)]

Risk factor		B	SE	OR (95%CI)	p value
Step 1^a^	Age	0.157	0.041	1.17 (1.08-1.27)	<0.001*
	Sex	0.761	0.603	2.14	0.207
	Education level	1.06	0.871	2.89	0.223
	Income	0	0	1	0.685
	Smoking	-0.146	0.828	0.86	0.86
	BMI	-0.015	0.074	0.99	0.837
	PISA	-0.001	0.001	0.99	0.152
	Constant	-9.038	30345	0	0.007

a Variables entered on step 1: age, sex, education level, income, smoking, body mass index (BMI), periodontal inflamed surface area (PISA) (Hosmer-Lemeshow test, p=0.150). SE=standard error

* Significant association with age p<0.001

## Discussion

In this cross-sectional study, the effect of the magnitude of the periodontal inflamed area on the serum APR and pro-/anti-inflammatory cytokines/ mediators in patients with/without CAD was evaluated. The investigated parameters (IL-1, IL-6, IL-10, TNF-α, SAA, PTX 3, hs-CRP) were not statistically significantly different between the study groups. Significant positive correlations between PISA scores and APR (SAA and PTX3) in CAD (-) groups (Groups 3 and 4) were observed. Multiple regression analysis revealed that only age has the predictability of having CAD among the investigated variables in this study groups.

The clarification of the mechanisms in the relationship between periodontitis and CAD has been matter of focus of studies in the last few decades, leading to suggestion of some plausible mechanisms^30^. However, the results of the studies in the existing literature should be cautiously evaluated to define a relationship between the diseases, due to the variety of the investigated population, their characteristics, methodology and investigated parameters (especially parameters evaluated after periodontal therapy)^22^.

This study is the first to quantitatively measure the periodontal inflamed surface area (PISA and PESA scores) in CAD patients regarding correlations with the circulating APR and cytokines. There has been a limited number of studies which have evaluated the association of PISA and PESA values with the systemic diseases, such as diabetes mellitus, and kidney functions^19^ ^,^ ^31^. In our study, the periodontal parameter values and PISA and PESA values were higher in periodontitis patients with/without CAD than in patients without periodontitis and with/without CAD, which was not a surprising result, since they were calculated using the PD, clinical attachment loss and BOP values. Although the serum cytokine and APR levels were not statistically significant between study groups, the positive correlations between the PISA values and SAA and PTX3 in CAD (-) groups with/ without periodontitis deserve further attention.

Ardila, et al.^4^ (2015) have found significantly elevated levels of SAA and CRP in periodontitis when compared to healthy controls. However, their study groups showed significant differences regarding triglycerides and high-density lipoproteins. It is known that cardiovascular diseases and related acute-phase reactions with elevated levels of CRP and SAA share various common risk factors involving smoking, high BMI, and older age^10^. In this study, the study groups were not statistically significantly different regarding blood lipid levels, BMI, and smoking status (Table 1). The correlations mentioned were found in CAD (-) patients who had an age lower than the CAD (+) groups. Despite the younger age in the CAD (-) patients, SAA has shown a significant correlation with PISA values, suggesting that the periodontal status might have affected the serum SAA levels. Adjustment for age does not alter the statistical comparative results; there was no significant difference regarding the circulating cytokines and APR between the study groups (p>0.0125, data not shown). However, multivariate logistic regression analysis revealed that only age has the predictability to having CAD among the investigated parameters in this study (Table 5). Our regression model was good fitted when the factors such as smoking, education, income and BMI were included (p=0.150). In a study conducted with an older population, it was suggested that the change in CRP levels with age depends mainly on the socioeconomic profile (SEP) which is mediated by metabolic alterations and health-risk behavior rather than age only^9^. After adjustments for age, sex, acute infection and chronic inflammatory conditions, very high CRP was associated with lower social position, depressive symptoms, physical inactivity, smoking, and alcohol abstinence in a geriatric population^16^. Our study groups were not significantly different when we evaluated factors regarding education level, income, smoking and BMI (p>0.0125). Further studies are needed to clarify the effect of SEP in the relationship between periodontitis and CAD regarding APR.

Pradeep, et al.^28^ (2011) have observed that the PTX3 level is higher in periodontitis patients. In fact, Gümüş, et al.^15^ (2014) suggested that PTX3 might be used as a diagnostic marker for periodontal inflammation. Nerkiz, et al.^25^ (2015) also reported that PTX3 levels may be regarded as a novel diagnostic predictor for CAD, and found higher levels in CAD (+) patients when compared to patients with normal coronary arteries. The positive correlations of PTX3 in CAD (-) patients (but referred to the coronary angiography with the suspicion of CAD) should be investigated with further longitudinal and interventional studies, taking into consideration the association of the SAA and PTX3 with CAD. Inflammation regarding periodontitis should be noted in patients with CAD and in patients at risk of further CAD, when the prevention of CAD was planned to include the periodontal treatment.

Another strength of the our study is that the study population was referred by the Cardiology Clinic; the study population is, therefore, a sample of CAD patients. The population selection was in accordance with the recommendations of the European Federation of Periodontology and American Academy of Periodontology to be recruited from medical offices rather than dental offices^32^. The cardiovascular status regarding CAD was verified using angiography, and after that evaluation, the groups were created considering the periodontal examination results. Tonetti, et al.^32^ (2013) also recommended that study populations should have substantial gingival inflammation (for example, bleeding on probing or PISA scoring system) and/or well-defined periodontal destruction for investigations regarding the relationship between periodontitis and CAD, to provide homogeneity of cases. In this study, the periodontal status was determined before coronary angiography and showed full mouth measurements (PD, clinical attachmet loss and BOP, which were used also to calculate the PISA and PESA) restrained the under/ over estimation of the periodontal status.

None of the serum cytokine/mediator levels were statistically significantly different between the study groups our study. Possible explanations include the fact that the study groups had mild, moderate and severe periodontitis patients (Table 2, Group 1 and Group 3). Vrazic, et al.^33^ (2015), and Zhu, et al.^34^ (2015) designed similar studies with similar patient groups. All studies showed significantly higher levels of the investigated parameters (IL-Ιβ, fibrinogen, CRP, etc.)^33^ ^,^ ^34^ and significant associations between the presence of coronary heart disease or acute coronary syndrome with the clinical periodontal parameters^30^. However, all study groups had severe periodontitis^33^ ^,^ ^34^. In this study, patients were grouped as having periodontitis or not. All patients without periodontitis were gingivitis patients with reduced periodontium^24^ (Group 2 and Group 4). There were 10 and 16 severe, 6 and 4 moderate, and 4 and 1 mild periodontitis^12^ patients in the periodontitis groups with CAD and without CAD, respectively (Groups 1 and Group 3). This evaluation was made after the study had been conducted, because the hypothesis was based on the presence of periodontitis and its impact (local inflamed area) on circulating APR in CAD patients. If the periodontitis groups had only severe periodontitis patients, the serum inflammatory markers would have been significantly different; Beck, et al.^7^ (2001) suggested that patients with severe generalized periodontitis had a higher risk of atheroma formation than those with less widespread disease, considering the dose-dependent effect of exposure. The size of each study group (periodontitis groups with and without CAD) was not sufficient to compare the different periodontitis statuses with each other in this study. Besides, the gingivitis status of the non-periodontitis groups (Group 2 and Group 4) has resulted in similar PISA and PESA scores (median: 28.13-427.26) as moderate periodontitis patients (median: 200.5-449.4) which was classified in accordance with the Centers for Disease Control and Prevention (CDC) and American Academy of Periodontology (AAP) case classifications by Sakanaka, et al.^29^ (2017). However, to the best of our knowledge, there were no studies which have evaluated gingivitis patients and their PISA and PESA scores to enable comments about gingivitis and inflamed surface areas. To understand this relationship further studies are warranted.

A second explanation might be the drugs used by CAD (+) patients. All CAD (+) patients were selected from the patients who use ACE inhibitors as an antihypertensive drug for this study, rather than calcium channel blockers, because there is more knowledge of their gingival enlargement effects^14^. Angiotensin-converting enzyme (ACE) inhibitors were reported to have an anti-inflammatory effect, and the usage of the drugs was reported to cause a decrease in the production of IL-1, IL-6, TNF-α, IL-8, CRP, IL- 12, interferon-γ, E-selectin, intracellular adhesion molecule-1, vascular cell adhesion molecule-1, monocyte chemoattractant molecule-1, and matrix metalloproteinase 9, along with the increased production of IL-10^21^. The positive correlation between IL-10 and PD in the entire study group (N = 77) was surprising when the anti-inflammatory role of IL-10 in periodontal disease was considered. However, IL- 10 seems to be a protective factor in atherosclerosis against the effects of pathogens^33^. Also, it is known that increased serum IL-10 levels are associated with a positive prognosis in acute coronary syndrome patients^8^. The increase in IL-10 in myocardial infarct patients was found to be correlated with systemic pro-inflammatory activity evaluated with thrombosis, plaque rupture and heart destruction related to IL-6 and TNF-α plasma concentrations^3^. Bing, et al.^8^ (2015) found lower levels of IL-10 in healthy patients when compared to patients with CAD (+) and periodontitis and patients with only periodontitis without CAD. Therefore, the positive correlation between PD and IL-10 in the whole study group is an important finding. This may be the result of the effort with the increased synthesis (levels) of IL-10 to protect/prevent atherosclerosis in the whole study group in this study, which had patients with coronary artery symptoms and a potential diagnosis of CAD.

Besides the aforementioned strengths, the cross-sectional nature of our study complicates the comments about the effect of periodontitis in the development and progression of CAD, in other words, in terms of causality, which might be considered as a limitation. Another limitation was the small sample size, although we have determined the sample size for each group before the study.

## Conclusion

When summarized, this study has revealed an important correlation within its limitations. The results of our study should be evaluated as preliminary. However, when the strengths of this study were considered, it can be suggested that our study has driven some APR (PTX3 and SAA) forward in the relationship between CAD and periodontitis. This should be investigated in more detail in interventional studies to clarify the relationship between periodontitis and CAD, and to develop strategies in the primary and secondary prevention of CAD.
